# S-Ketamine Pretreatment Alleviates Anxiety-Like Behaviors and Mechanical Allodynia and Blocks the Pro-inflammatory Response in Striatum and Periaqueductal Gray From a Post-traumatic Stress Disorder Model

**DOI:** 10.3389/fnbeh.2022.848232

**Published:** 2022-04-14

**Authors:** Shuai Yang, Ke Xu, Xuan Xu, Jixiang Zhu, Yinan Jin, Qi Liu, Rui Xu, Xiaoping Gu, Yue Liu, Yulin Huang, Zhengliang Ma

**Affiliations:** Department of Anesthesiology, Affiliated Drum Tower Hospital of Medical Department, Nanjing University, Nanjing, China

**Keywords:** post-traumatic stress disorder, single prolonged stress, microglia, S-ketamine, pro-inflammatory cytokines

## Abstract

This study aims to explore the regulatory effect of S-ketamine on the mechanical allodynia, anxiety-like behaviors and microglia activation in adult male rats exposed to an animal model of post-traumatic stress disorder (PTSD). The rat PTSD model was established by the exposure to single-prolonged stress (SPS), and 1 day later, rats were intraperitoneally injected with 5 mg/kg S-ketamine or normal saline, respectively. Paw withdrawal mechanical threshold was measured 2 days before, and 1, 3, 5, 7, 10, 14, 21 and 28 days after injection to assess mechanical allodynia in the SPS-exposed rats. For anxiety-like behaviors, the open field test and elevated plus maze test were performed at 7 and 14 days after S-ketamine treatment in the SPS-exposed rats, respectively. SPS-induced rats presented pronounced mechanical allodynia and anxiety-like behaviors, which were alleviated by S-ketamine treatment. After behavioral tests, rats were sacrificed for collecting the anterior cingulate cortex (ACC), prefrontal cortex (PFC), dorsal striatum, and periaqueductal gray (PAG). Protein levels of TNF-α, IL-1β, p-NF-κB, and NF-κB in brain regions were examined by Western blot. In addition, microglia activation in each brain region was determined by immunofluorescence staining of the microglia-specific biomarker Iba-1. Interestingly, pro-inflammatory cytokines were significantly upregulated in the dorsal striatum and PAG, rather than ACC and PFC. Activated microglia was observed in the dorsal striatum and PAG as well, and upregulated p-NF-κB was detected in the dorsal striatum. Inflammatory response, phosphorylation of NF-κB and microglia activation in certain brain regions were significantly alleviated by S-ketamine treatment. Collectively, S-ketamine is a promising drug in alleviating mechanical allodynia, anxiety-like behaviors, and pro-inflammatory responses in discrete brain regions in a model of PTSD.

## Introduction

Post-traumatic stress disorder (PTSD) is a chronic and psychological disorder that develops after experiencing extreme traumatic events (Liu et al., 2017). PTSD patients usually suffer recurrent and invasive traumatic memories, leading to severe anxiety (Bisson, 2007). A positive correlation between PTSD-induced anxiety and chronic pain has been validated, and approximately 50% of patients have a declined quality of life due to the chronic pain (Barbano et al., 2021; Gasperi et al., 2021). The co-existence of anxiety and pain could worse outcomes for both disorders and may reduce treatment response (Zhuo, 2016). However, to our knowledge, neurobiological data supporting the interaction between anxiety and mechanical allodynia in PTSD cases are scant. So far, targeted treatment is limited and urgently needed.

Microglia are innate immune cells in the central nervous system (CNS) that express multiple receptors responsive to stress hormones like glucocorticoids and catecholamines (Frank et al., 2015; Weber et al., 2017). The morphology and function of microglia are mediated by various physical and psychological stimuli, which in turn provide an inflammatory microenvironment in affective brain regions (e.g., striatum and amygdala) (Frank et al., 2019; Munshi et al., 2020; Cheng et al., 2021). Morphological changes of microglia commonly occurred in anxiety, stress, and other psychiatric disorders (Caetano et al., 2017; Adeluyi et al., 2019; Liu et al., 2022). Activated microglia were observed by PET imaging examination in different brain areas, such as prefrontal cortex (PFC), anterior cingulate cortex (ACC), hippocampus, and amygdala (Setiawan et al., 2015). Dysregulated activation of microglia in these brain regions are responsible for the presence of anxiety-like behaviors by upregulating pro-inflammatory cytokines like interleukin-1β (IL-1β) and tumor necrosis factor-α (TNF-α) (Han et al., 2020; Cotrone et al., 2021; Zheng et al., 2021), which are known as potent neuromodulators for synaptic transmission *via* the microglia-neuron crosstalk (Chen et al., 2018). In addition to processing anxiety information, the striatum and amygdala are also involved in the formation of pain (Corder et al., 2019; Arora et al., 2021). Pain transmission is regulated by descending pathways originating in the brainstem and in other cerebral structures [e.g., periaqueductal gray (PAG), ACC, and hypothalamus], which modulate the nociceptive information at the spinal dorsal horn mainly by reducing the release of neurotransmitters from primary afferent neurons or by inhibiting projection neurons and excitatory interneurons (Millan, 1999; Tsuda et al., 2017). The activation of descending pain modulatory circuits from the PAG is one of the potential causes of chronic pain (Leith et al., 2010). It is reported that the abnormal activation of microglia in the ventrolateral PAG may accelerate descending facilitation in a constriction nerve injury model (Ni et al., 2016). Inhibition of microglia activation in these brain regions can alleviate rat anxiety-like behaviors or hyperalgesia. Consistently, our previous findings proved the abnormal activation of microglia in the spinal cord and hippocampus of rats exposed to the single-prolonged stress (SPS) (Sun et al., 2016, 2017).

The clinical benefits of (R,S)-ketamine in treat-resistant PTSD have been previously validated, which significantly improve negative emotions (Feder et al., 2014, 2021). Moreover, (R,S)-ketamine is reported to reduce anxiety-like behaviors in mice subjected to the electric foot shock (Zhang et al., 2015). The anti-inflammatory and immunomodulatory effects of (R,S)-ketamine in the CNS have been demonstrated as well (Dominguini et al., 2021). An *in vitro* experiment revealed that (R,S)-ketamine downregulates pro-inflammatory cytokines by inhibiting the activation of nuclear factor-kappa B (NF-κB) in lipopolysaccharides (LPS)-induced BV2 cells (Lu et al., 2020). Compared with those of (R,S)-ketamine and R-ketamine, the ketamine enantiomer S-ketamine is much safer with fewer neurological complications. In addition to the anti-depression effect, S-ketamine can also be applied as analgesic and anti-anxiety drug (Hayashi et al., 2011; Maraschin et al., 2021). The anti-inflammatory function of S-ketamine has been reported in a neuropathic pain model through alleviating microglia activation in the spinal cord (Hayashi et al., 2011). The potential influence of S-ketamine on PTSD-induced anxiety, mechanical allodynia, and microglia activation, however, remain unclear.

In the present study, we established a rat PTSD model by SPS to identify the biological function of S-ketamine treatment on alleviating anxiety-like behaviors, pain, and activation of microglia in different brain regions.

## Materials and Methods

### The Rat Post-traumatic Stress Disorder Model

Adult male Sprague-Dawley (SD) rats weighing 200–250 g provided by the Laboratory Animal Center of Nanjing Drum Tower Hospital were habituated at controlled temperature (21 ± 1°C) with an alternating 12/12 h light/dark cycle for 1 week. They had free access to food and water. *In vivo* experiments were approved by the Policy on the Use of Animals in Nanjing Drum Tower Hospital and were conducted according to the ethical guidelines of the International Association for the Study of Pain (IASP).

The PTSD model was prepared by SPS as previously described (Liberzon et al., 1997). Briefly, rats were restrained in a plastic animal holder for 2 h, immediately forced to 20-min group swimming in a transparent cylindrical glass containers (50 cm in height, 20 cm in diameter, one rat in a glass container at a time) filled with 24°C water, rested for 15 min, and exposed to sevoflurane until loss of consciousness. Rats in control group were placed in the adjacent room without specific treatment. One day after SPS, rats in PTSD group and control group were intraperitoneally injected with 5 mg/kg S-ketamine (200325BL, Jiangsu Hengrui Pharmaceuticals, China) or normal saline, respectively.

### Behavioral Tests

#### Paw Withdrawal Mechanical Threshold

Paw withdrawal mechanical threshold (PWMT) was measured 2 days before S-ketamine injection, and 1, 3, 5, 7, 10, 14, 21, and 28 days after injection, aiming to reveal mechanical allodynia (Sun et al., 2019). Behavioral tests are performed at the same time point at the day per individual experiment in the same room. Experimenters were well trained to perform the tests and were blinded for the experimental groups. PWMT were assessed with the dynamic plantar esthesiometer (No. 37450, UGO BASILE, Italy) Prior to the start of experiments, rats were acclimatized to the testing environment by placing the rats on elevated mesh screen in individual Plexiglas chambers for at least 30 min. PWMT was recorded for five times, with a minimum interval of 10 min. The metal wire was pressed vertically against the center of the rat’s right hind paws, and an upward pressure gradually increased from 1 to 50 g. PWMT was recorded as the tolerance level in grams, and the mean PWMT was calculated by averaging the five tests.

#### Open Field Test and Elevated Plus Maze Test

Animal trajectory tracking system (Etho Vision XT 11.5, Noldus, Netherlands) was performed to collect the data of open field test (OFT) and elevated plus maze test (EPMT) in this study.

Exploratory activities and anxiety-like behaviors of rats were assessed by the OFT (Hiroi and Neumaier, 2006) on the seventh day in an open box (80 cm × 40 cm) consisted of opaque acrylic plates, in which the bottom was divided into the central region and edge region. During the test, the rat was initially placed in the corner of open box, and allowed for 10-min exploration behaviors recorded by a camera, and the distanced traveled and entries in the center were analyzed using the video tracking system.

The EPMT was performed on the 14th day to assess anxiety-like behaviors as previously described (Walf and Frye, 2007). The elevated apparatus at 50 cm above the floor consisted of four arms, including two opposite open arms (50 cm × 10 cm) and two closed arms (50 cm × 10 cm × 40 cm). During the test, the rat was placed in the central area facing an open arm, and allowed for 5-min exploration behaviors recorded by a camera. Anxiety index were calculated on the basis of 1-[(time spent in open arm / total time on the maze) / 2 + (number of entries to the open arms / total exploration on the maze) / 2] as defined in previous study (Serova et al., 2013). Total exploration was calculated as the number of entries into any arm of the maze in order to distinguish between impaired exploratory behavior, exploration limited to closed arms (avoidance), and free exploration (Cohen et al., 2008).

### Western Blotting

Rats were sacrificed by cervical dislocation method after sevoflurane anesthesia, followed with removing skull for taking fresh brain sections on the 14th day. Different fresh brain region tissues were collected by cutting in a rat brain mold (68715, RWD, China) and stored at −80°C for further protein extraction. Briefly, the whole rat brain was placed correctly according to the shape of the brain mold in the upward direction of the skull base. Subsequently, the rat brain in the brain mold was inserted with the blades from the optic chiasm as the starting point and cut piece by piece into the groove gap of the brain mold at an interval of 1 mm. The corresponding brain regions were isolated according to the position of each brain region in the brain atlas. The brain tissues were homogenized in lysis buffer followed by centrifugation at 12,000 rpm for 20 min at 4°C. Protein supernatant of each samples were quantified to 2 μg/μl then 20 μg protein in total per sample were separated by 10% SDS-PAGE and loaded on polyvinylidene fluoride (PVDF) membranes. The filter membranes were blocked with 5% non-fat milk in tris buffered saline with Tween20 (TBST) buffer for 1 h at room temperature and incubated with the anti-IL-1β (1:1,000, ab9722, Abcam), anti-TNF-α (1:200, sc-52746, Santa Crus), anti-NF-κB (1:1,000, 8242, Cell Signaling Technology), anti-p-NF-κB (1:1,000, 3033, Cell Signaling Technology) and anti-β-actin antibody (1:10,000, bs-0061R, Bioss) at 4°C overnight. After washed with TBST for five times (5 min for each time), the filter membranes were incubated with corresponding secondary antibodies (HRP-conjugated goat anti-rabbit, 1:20,000, #A0545, Millipore; HRP-conjugated goat anti-mouse, 1:20,000, #A9044, Millipore) at room temperature for 1 h and washed with TBST for three times (10 min for each time) afterward. Band exposure was achieved by the enhanced chemiluminescence method (4600SF, Tanon, China). ImageJ software (NIH, Bethesda, MD, United States) was used to analyze the band intensity.

### Immunofluorescence

After cardiac perfusion of saline and 4% paraformaldehyde (PFA), rat brains were collected, fixed in 4% PFA for 24 h, and then dehydrated in 0.1 M phosphate buffered saline (PBS) containing 20 and 30% sucrose at 4°C for 24 and 48 h, respectively. Fixed tissues containing ACC/PFC (Bregma 3.70 to 2.70 mm), PAG (Bregma −6.24 to −6.60 mm), and the dorsal striatum (Bregma 1.44 to 1.08 mm) were frozen and sliced into 20 μm slides using a freezing microtome according to the sixth Rat Brain in Stereotaxic Coordinates (Paxinos and Watson, 2006).

After PBS wash and 1-h blockage of non-specific antigens containing 10% goat serum with 0.3% Triton X-100 for 2 h at room temperature, the brain sections were incubated with the primary antibody (anti-Iba1 antibody, 1:500, 019–19741, Wako) overnight at 4°C, and the secondary antibody (Alexa 488-conjugated goat anti-rabbit, 1:3,000, R37116, Life Technologies) for 2 h at room temperature. Sections were mounted on glass slides, incubated with DAPI (ab104139, Abcam) in the dark and captured through a laser-scanning confocal microscope (DP80, Olympus, Japan). Three of sections per animal were employed for further statistical analysis. ImageJ software (NIH, Bethesda, MD, United States) was used to analyze the fluorescent intensity.

### Statistical Analysis

SPSS 22.0 software was used for statistical analysis. All data were shown as the mean ± standard deviation (SD). Two-way repeated measures analysis of variance (ANOVA) followed by the Bonferroni *post hoc* test was carried out to compare the changes of PWMT data among groups at each time point and one-way ANOVA followed by Bonferroni test to assess differences over time within groups. For the data of Western blot, immunofluorescence and anxiety index were analyzed with 2 × 2 ANOVA followed by Bonferroni test between-group comparisons. *P* < 0.05 considered as statistically significant.

## Results

### S-Ketamine Alleviates Mechanical Allodynia in the Single-Prolonged Stress-Exposed Rats

**FIGURE 1:**
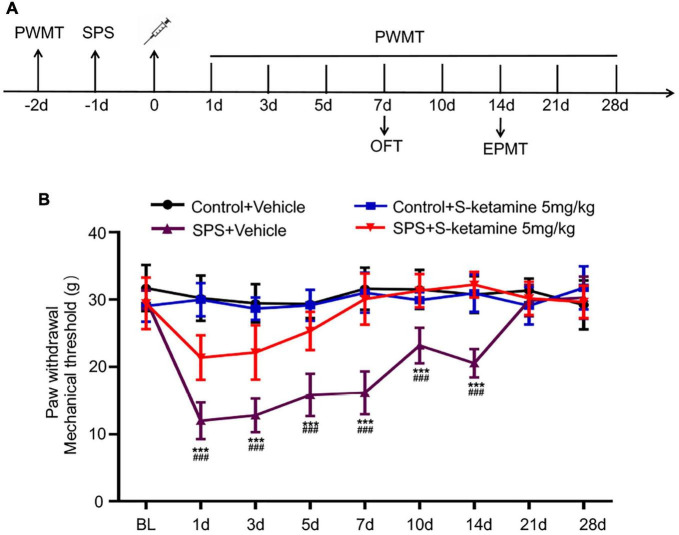
S-ketamine alleviates chronic pain in the SPS-exposed rats. **(A)** Scheme diagram of behavioral tests and SPS injection in rats. Rats were randomly assigned into control + vehicle group, SPS + vehicle group, control + S-ketamine 5 mg/kg group and SPS + S-ketamine 5 mg/kg group. *n* = 8 per group. **(B)** Paw withdrawal mechanical threshold of rats in each group. ***P < 0.001, SPS + vehicle group vs. control + vehicle group; ^###^*P* < 0.001, SPS + S-ketamine 5 mg/kg group vs. SPS + vehicle group.

In the present study, rats were exposed to SPS and on the next day, they were respectively peritoneally administrated with 5 mg/kg S-ketamine or saline. PWMT was first performed 2 days before S-ketamine administration, and 1, 3, 5, 7, 10, 14, 21, and 28 days after administration, respectively, aiming to reveal nociceptive behaviors in the SPS-exposed rats (Figure 1A). PWMT prior to SPS was comparable among the four groups. It is shown that compared with those administrated with vehicle, SPS exposure significantly reduced PWMT at 1, 3, 5, 7, 10, and 14 days (*F*_(3,21)_ = 134.825, *p* < 0.001 for group factor, *F*_(8,56)_ = 30.350, *p* < 0.001 for time factor, *F*_(24,168)_ = 15.225, *p* < 0.001 for interaction. Control + vehicle group vs. SPS + vehicle group: *p* < 0.001 for all time points; SPS + vehicle group vs. SPS + S-ketamine 5 mg/kg group: *p* < 0.001 for all time points. Day1: *F*_(3,21)_ = 87.254, *p* < 0.001 for group factor; Day3: *F*_(3,21)_ = 43.114, *p* < 0.001 for group factor; Day5: *F*_(3,21)_ = 46.893, *p* < 0.001 for group factor; Day7: *F*_(3,21)_ = 54.652, *p* < 0.001 for group factor; Day10: *F*_(3,21)_ = 24.317, *p* < 0.001 for group factor; Day14: *F*_(3,21)_ = 41.507, *p* < 0.001 for group factor) (Figure 1B). It is suggested that S-ketamine effectively ameliorated mechanical allodynia in the SPS-exposed rats.

### S-Ketamine Alleviates Anxiety-Like Behaviors in the Single-Prolonged Stress-Exposed Rats

**FIGURE 2:**
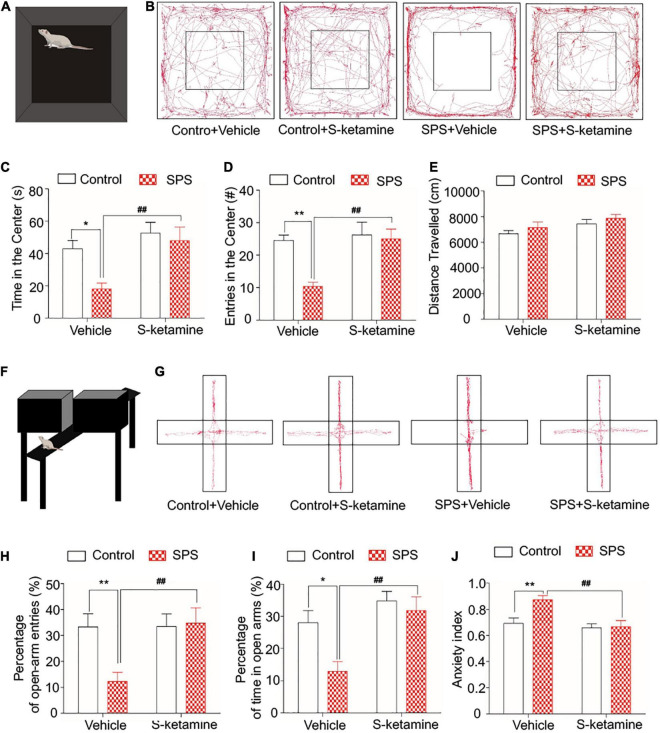
S-ketamine alleviates anxiety-like behaviors in the SPS-exposed rats. Rats were randomly assigned into control + vehicle group, SPS + vehicle group, control + S-ketamine 5 mg/kg group and SPS + S-ketamine 5 mg/kg group. *n* = 8 per group. **(A)** Diagram of the open field test. **(B)** Movement paths in the open field. **(C)** Time in the center. **(D)** Entries in the center. **(E)** Distance traveled. **(F)** Diagram of the elevated plus maze test. **(G)** Movement paths in the open and closed arms. **(H)** Percentage of open-arm entries. **(I)** Percentage of time in open arms. **(J)** Anxiety index. *P < 0.05, **P < 0.01, control + vehicle group vs. SPS + vehicle group; ^#^*P* < 0.05, ^##^*P* < 0.01, SPS + S-ketamine 5 mg/kg group vs. SPS + vehicle group.

Open field test and EPMT were performed to test anxiety-like behaviors in the SPS-exposed rats. The movement of rats was visualized in Figures 2A,B. Rats that received SPS + vehicle showed anxiety-like behavior in the OFT, manifesting as significantly reduced time in the center and the frequency of entries in the center. Notably, S-ketamine treatment remarkably elevated them, indicating its function in alleviating anxiety-like behaviors in the SPS-exposed rats (time in the center: *F*_(1,28)_ = 5.704, *p* = 0.024 for group factor, *F*_(1,28)_ = 10.105, *p* = 0.004 for drug factor, *F*_(1,28)_ = 2.701, *p* > 0.05 for interaction; frequency of entries in the center: *F*_(1,28)_ = 13.664, *p* = 0.001 for group factor, *F*_(1,28)_ = 14.648, *p* = 0.001 for drug factor, *F*_(1,28)_ = 5.676, *p* = 0.024 for interaction) (Figures 2C,D). However, no significant difference in the distance traveled was detected before and after S-ketamine treatment (*p* > 0.05) (Figure 2E). In addition, EPMT was performed using the cross-shaped apparatus (Figures 2F,G). SPS-induced rats showed a significant decrease in time spent and entries in open arms as well as the anxiety index, indicating anxiety-like behaviors. S-ketamine treatment significantly increased the percentage of open-arm entries (*F*_(1,28)_ = 8.968, *p* = 0.006 for group factor, *F*_(1,28)_ = 10.512, *p* = 0.003 for drug factor, *F*_(1,28)_ = 5.198, *p* = 0.03 for interaction) (Figure 2H) and time spent in open arms (*F*_(1,28)_ = 6.432, *p* = 0.017 for group factor, *F*_(1,28)_ = 12.850, *p* = 0.001 for drug factor, *F*_(1,28)_ = 2.887, *p* > 0.05 for interaction) (Figure 2I), and reduced the anxiety index (*F*_(1,28)_ = 11.019, *p* = 0.003 for group factor, *F*_(1,28)_ = 14.434, *p* = 0.001 for drug factor, *F*_(1,28)_ = 11.019, *p* = 0.33 for interaction) (Figure 2J). Taken together, S-ketamine effectively alleviated SPS-induced anxiety-like behaviors.

### S-Ketamine Does Not Influence Microglial Activation and Pro-inflammatory Cytokines in the Anterior Cingulate Cortex and Prefrontal Cortex

**FIGURE 3:**
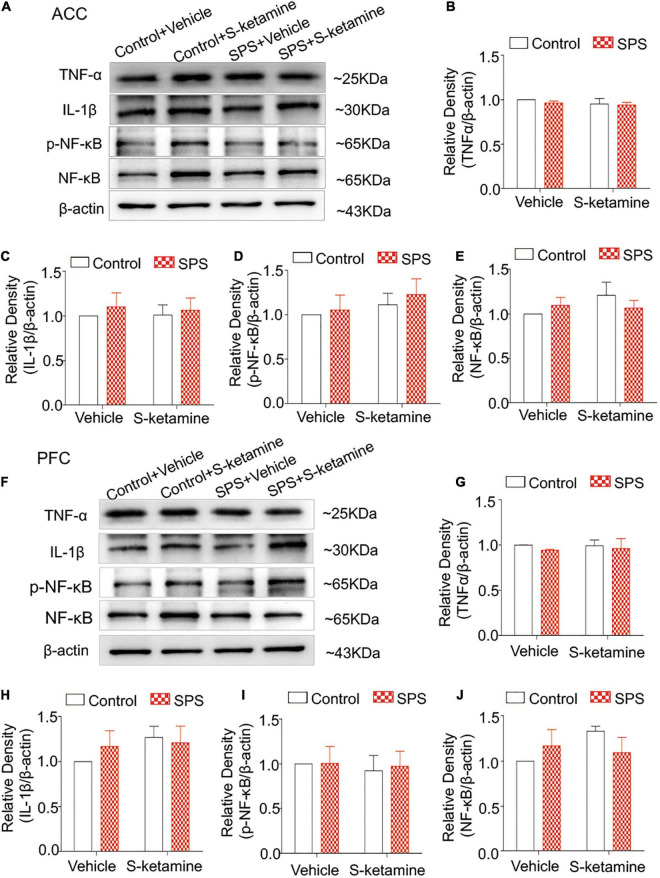
S-ketamine does not influence pro-inflammatory cytokines in the anterior cingulate cortex and prefrontal cortex. Rats were randomly assigned into control + vehicle group, SPS + vehicle group, control + S-ketamine 5 mg/kg group and SPS + S-ketamine 5 mg/kg group. *n* = 3 per group. **(A)** Protein levels of TNF-α, IL-1β, p-NF-κB, and NF-κB in the ACC. Relative intensity of TNF-α **(B)**, IL-1β **(C)**, p-NF-κB **(D)**, and NF-κB **(E)** in the ACC. **(F)** Protein levels of TNF-α, IL-1β, p-NF-κB, and NF-κB in the PFC. Relative intensity of TNF-α **(G)**, IL-1β **(H)**, p-NF-κB **(I)**, and NF-κB **(J)** in the PFC.

Inflammatory response is mediated by microglia. Here, we detected protein levels of TNF-α, IL-1β, p-NF-κB, and NF-κB in brain regions were examined by Western blot. Typical brain regions of rats were collected after sacrifice, including the ACC, PFC, the dorsal striatum, and PAG. However, we did not detect significant differences in their protein levels in the ACC of rats exposed to SPS or saline. S-ketamine treatment did not change their levels (protein levels of TNF-α: *F*_(1,8)_ = 0.401, *p* > 0.05 for group factor, *F*_(1,8)_ = 0.939, *p* > 0.05 for drug factor, *F*_(1,8)_ = 0.125, *p* > 0.05 for interaction; protein levels of IL-1β: *F*_(1,8)_ = 0.438, *p* > 0.05 for group factor, *F*_(1,8)_ = 0.014, *p* > 0.05 for drug factor, *F*_(1,8)_ = 0.045, *p* > 0.05 for interaction; protein levels of p-NF-κB: *F*_(1,8)_ = 0.369, *p* > 0.05 for group factor, *F*_(1,8)_ = 1.060, *p* > 0.05 for drug factor, *F*_(1,8)_ = 0.051, *p* > 0.05 for interaction; protein levels of NF-κB: *F*_(1,8)_ = 0.058, *p* > 0.05 for group factor, *F*_(1,8)_ = 0.839, *p* > 0.05 for drug factor, *F*_(1,8)_ = 1.490, *p* > 0.05 for interaction) (Figures 3A–E). Similarly, their protein levels were not changed in PAG as well (protein levels of TNF-α: *F*_(1,8)_ = 0.476, *p* > 0.05 for group factor, *F*_(1,8)_ = 0.06, *p* > 0.05 for drug factor, *F*_(1,8)_ = 0.045, *p* > 0.05 for interaction; protein levels of IL-1β: *F*_(1,8)_ = 0.141, *p* > 0.05 for group factor, *F*_(1,8)_ = 1.148, *p* > 0.05 for drug factor, *F*_(1,8)_ = 0.625, *p* > 0.05 for interaction; protein levels of p-NF-κB: *F*_(1,8)_ = 0.036, *p* > 0.05 for group factor, *F*_(1,8)_ = 0.132, *p* > 0.05 for drug factor, *F*_(1,8)_ = 0.024, *p* > 0.05 for interaction; protein levels of NF-κB: *F*_(1,8)_ = 0.077, *p* > 0.05 for group factor, *F*_(1,8)_ = 1.013, *p* > 0.05 for drug factor, *F*_(1,8)_ = 2.579, *p* > 0.05 for interaction) (Figures 3F–J).

**FIGURE 4:**
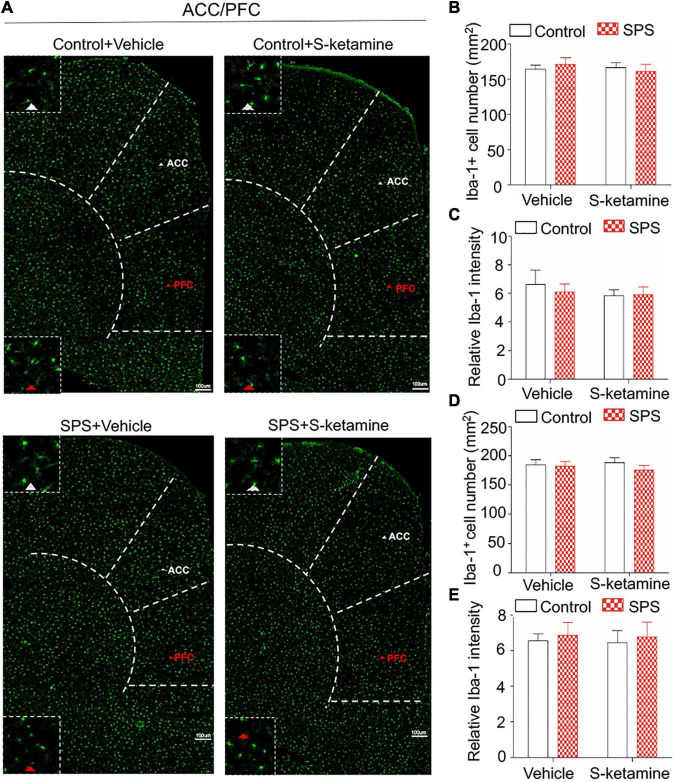
S-ketamine does not influence the microglial activation in the anterior cingulate cortex and prefrontal cortex. Rats were randomly assigned into control + vehicle group, SPS + vehicle group, control + S-ketamine 5 mg/kg group and SPS + S-ketamine 5 mg/kg group. *n* = 3 per group. **(A)** Immunofluorescence staining of Iba-1 in ACC and PFC. **(B)** The number of Iba-1-positive stained cells in the ACC. **(C)** Relative intensity of Iba-1-positive stained cells in the ACC. **(D)** The number of Iba-1-positive stained cells in the PFC. **(E)** Relative intensity of Iba-1-positive stained cells in the PFC.

Microglia activation was tested by immunofluorescence staining of Iba-1, and we calculated the number of Iba-1-positive stained cells and its relative intensity (Figure 4A). No significant difference in the positive staining of Iba-1 was observed in the ACC (number of Iba-1-positive cells: *F*_(1,8)_ = 0.010, *p* > 0.05 for group factor, *F*_(1,8)_ = 0.215, *p* > 0.05 for drug factor, *F*_(1,8)_ = 0.058, *p* > 0.05 for interaction; relative intensity: *F*_(1,8)_ = 0.121, *p* > 0.05 for group factor, *F*_(1,8)_ = 0.521, *p* > 0.05 for drug factor, *F*_(1,8)_ = 0.202, *p* > 0.05 for interaction) (Figures 4B,C) and PFC (number of Iba-1-positive cells: *F*_(1,8)_ = 0.806, *p* > 0.05 for group factor, *F*_(1,8)_ = 0.020, *p* > 0.05 for drug factor, *F*_(1,8)_ = 0.383, *p* > 0.05 for interaction; relative intensity: *F*_(1,8)_ = 0.229, *p* > 0.05 for group factor, *F*_(1,8)_ = 0.022, *p* > 0.05 for drug factor, *F*_(1,8)_ = 0.001, *p* > 0.05 for interaction) (Figures 4D,E) of rats exposed to SPS or saline. S-ketamine treatment did not influence microglia activation in the two brain regions (Figures 4B–E).

### S-Ketamine Alleviates Microglial Activation and Downregulates Pro-inflammatory Cytokines in the Striatum

**FIGURE 5:**
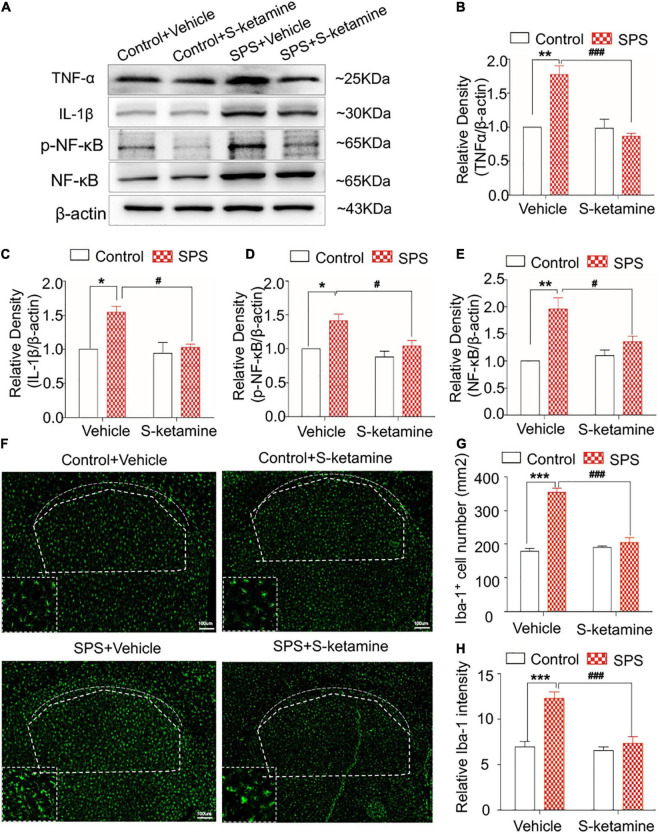
S-ketamine alleviates microglial activation and downregulates pro-inflammatory cytokines in the dorsal striatum. Rats were randomly assigned into control + vehicle group, SPS + vehicle group, control + S-ketamine 5 mg/kg group and SPS + S-ketamine 5 mg/kg group. *n* = 3 per group. **(A)** Protein levels of TNF-α, IL-1β, p-NF-κB, and NF-κB in the dorsal striatum. Relative intensity of TNF-α **(B)**, IL-1β **(C)**, p-NF-κB **(D)**, and NF-κB **(E)** in the dorsal striatum. **(F)** Immunofluorescence staining of Iba-1 in the dorsal striatum. **(G)** The number of Iba-1-positive stained cells in the dorsal striatum. **(H)** Relative intensity of Iba-1-positive stained cells in the dorsal striatum. *P < 0.05, **P < 0.01, and ***P < 0.001, control + vehicle group vs. SPS + vehicle group; ^#^*P* < 0.05, ^###^*P* < 0.001, SPS + S-ketamine 5 mg/kg group vs. SPS + vehicle group.

We further examined inflammatory response and microglial activation in the striatum. Protein levels of TNF-α, IL-1β, p-NF-κB, and NF-κB were significantly upregulated in the dorsal striatum of SPS-induced rats, which were markedly reversed by S-ketamine treatment (protein levels of TNF-α: *F*_(1,8)_ = 24.704, *p* = 0.001 for group factor, *F*_(1,8)_ = 34.114, *p* < 0.001 for drug factor, *F*_(1,8)_ = 16.160, *p* = 0.004 for interaction; protein levels of IL-1β: *F*_(1,8)_ = 7.617, *p* = 0.025 for group factor, *F*_(1,8)_ = 6.780, *p* = 0.031 for drug factor, *F*_(1,8)_ = 4.448, *p* > 0.05 for interaction; protein levels of p-NF-κB: *F*_(1,8)_ = 10.133, *p* = 0.015 for group factor, *F*_(1,8)_ = 8.424, *p* = 0.027 for drug factor, *F*_(1,8)_ = 1.965, *p* > 0.05 for interaction; protein levels of NF-κB: *F*_(1,8)_ = 22.400, *p* = 0.001 for group factor, *F*_(1,8)_ = 8.658, *p* = 0.019 for drug factor, *F*_(1,8)_ = 5.878, *p* = 0.042 for interaction) (Figures 5A–E). Compared with those of controls, SPS exposure significantly enhanced Iba-1 staining in the striatum, indicating microglial activation in the striatum of the SPS-exposed rats. S-ketamine treatment effectively inhibited the number of Iba-1-positive cells and its relative intensity, suggesting the inhibited microglia activation (number of Iba-1-positive cells: *F*_(1,8)_ = 132.345, *p* < 0.001 for group factor, *F*_(1,8)_ = 96.131, *p* < 0.001 for drug factor, *F*_(1,8)_ = 55.833, *p* < 0.001 for interaction; relative intensity: *F*_(1,8)_ = 37.398, *p* < 0.001 for group factor, *F*_(1,8)_ = 32.333, *p* < 0.001 for drug factor, *F*_(1,8)_ = 13.610, *p* = 0.006 for interaction) (Figures 5F–H).

### S-Ketamine Alleviates Microglial Activation and Downregulates Pro-inflammatory Cytokines in the Periaqueductal Gray

**FIGURE 6:**
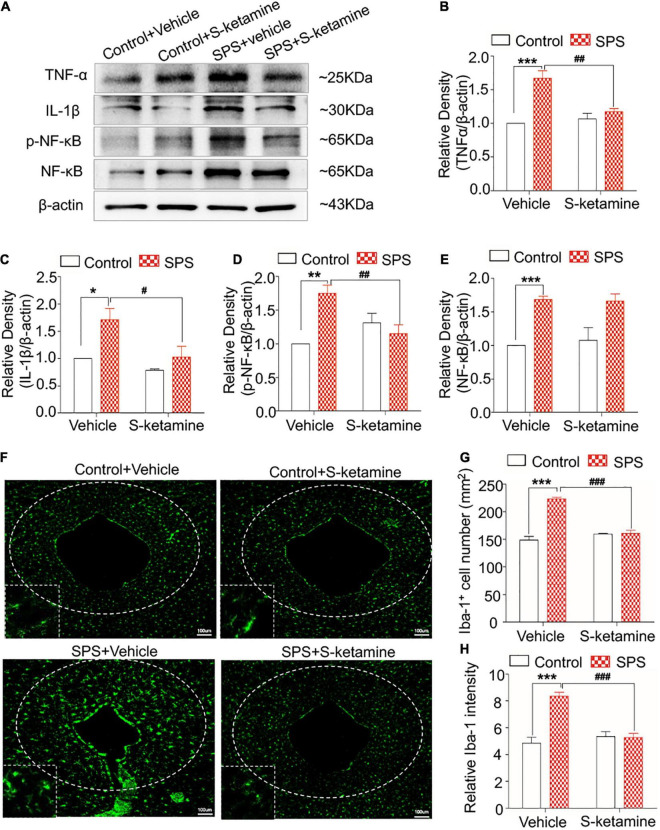
S-ketamine alleviates microglial activation and downregulates pro-inflammatory cytokines in the periaqueductal gray. Rats were randomly assigned into control + vehicle group, SPS + vehicle group, control + S-ketamine 5 mg/kg group and SPS + S-ketamine 5 mg/kg group. *n* = 3 per group. **(A)** Protein levels of TNF-α, IL-1β, p-NF-κB, and NF-κB in the PAG. Relative intensity of TNF-α **(B)**, IL-1β **(C)**, p-NF-κB **(D)**, and NF-κB **(E)** in the PAG. **(F)** Immunofluorescence staining of Iba-1 in the dorsal striatum. **(G)** The number of Iba-1-positive stained cells in the PAG. **(H)** Relative intensity of Iba-1-positive stained cells in the PAG. *P < 0.05, **P < 0.01, and ***P < 0.001, control + vehicle group vs. SPS + vehicle group; ^#^*P* < 0.05, ^##^*P* < 0.01, and ^###^*P* < 0.001, SPS + S-ketamine 5 mg/kg group vs. SPS + vehicle group.

Upregulated the expression of TNF-α, IL-1β, and p-NF-κB were identified in PAG of SPS-induced rats compared with control rats, and effectively reduced by S-ketamine treatment (protein levels of TNF-α: *F*_(1,8)_ = 41.715, *p* < 0.001 for group factor, *F*_(1,8)_ = 22.967, *p* = 0.001 for drug factor, *F*_(1,8)_ = 14.737, *p* = 0.005 for interaction; protein levels of IL-1β: *F*_(1,8)_ = 11.284, *p* = 0.01 for group factor, *F*_(1,8)_ = 11.487, *p* = 0.014 for drug factor, *F*_(1,8)_ = 2.708, *p* > 0.05 for interaction; protein levels of p-NF-κB: *F*_(1,8)_ = 21.597, *p* = 0.002 for group factor, *F*_(1,8)_ = 13.707, *p* = 0.006 for drug factor, *F*_(1,8)_ = 15.832, *p* = 0.004 for interaction; protein levels of NF-κB: *F*_(1,8)_ = 31.915, *p* < 0.001 for group factor, *F*_(1,8)_ = 0.056, *p* > 0.05 for drug factor, *F*_(1,8)_ = 0.0218, *p* > 0.05 for interaction) (Figures 6A–E). As expected, the activated and increased numbers of microglia in PAG following SPS exposure was alleviated by S-ketamine treatment (number of Iba-1-positive cells: *F*_(1,8)_ = 63.673, *p* < 0.001 for group factor, *F*_(1,8)_ = 44.297, *p* < 0.001 for drug factor, *F*_(1,8)_ = 30.705, *p* = 0.001 for interaction; relative intensity: *F*_(1,8)_ = 46.060, *p* < 0.001 for group factor, *F*_(1,8)_ = 35.622, *p* < 0.001 for drug factor, *F*_(1,8)_ = 24.103, *p* = 0.001 for interaction) (Figures 6F–H).

## Discussion

The current study demonstrated that SPS induced microglial activation and upregulated proinflammatory cytokines in the dorsal striatum and PAG, and those in the ACC and PFC were not influenced. Importantly, intraperitoneal injection of S-ketamine alleviated mechanical allodynia and anxiety-like behaviors, inhibited microglial activation and downregulated proinflammatory cytokine in the dorsal striatum and PAG in the SPS-exposed rats. These results suggested that S-ketamine is a promising drug for alleviating PTSD-induced anxiety and mechanical allodynia, as well as preventing the upregulation of microglia activation in certain brain regions.

We first established the rat PTSD model by the exposure to SPS. PTSD behaviors of SPS-induced rats were first examined through a series of behavioral tests. Compared with rats administrated with vehicle, SPS-induced rats presented significantly lower PWMT, time in the center, entries in the center, percentage of open-arm entries, percentage of time in open arms, and anxiety index. Chronic pain often accompanies anxiety, which enhances the sensitivity to pain and prolongs the recovery time. Chronic pain in turn aggravates anxiety symptoms, presenting a mutual interaction (Ji et al., 2021). Stress is believed to cause neuroendocrine dysfunction in corresponding brain regions, thus influencing the pain facilitation and inhibition. Nevertheless, commonly used anti-anxiety drugs usually have severe adverse events like peripheral nerve palsy and gastrointestinal dysfunction (Matisz and Gruber, 2022). A growing number of evidences have shown that the sub-anesthetic doses of ketamine not only exert good anti-anxiety and anti-depressive effects, but also alleviate hyperalgesia (Berrino et al., 2003; Petrocchi et al., 2019). *In vivo* study showed that low-dose ketamine alleviates anxiety-like behaviors in mice after electric shock (Zhang et al., 2015). A low-dose S-ketamine can relieve anxiety symptoms in palliative care patients, presenting a good analgesic effect (Falk et al., 2020). Consistently, our data revealed that peritoneal administration of 5 mg/kg S-ketamine effectively alleviated anxiety-like behaviors and chronic mechanical allodynia in the SPS-exposed rats.

Previous studies have shown that SPS exposure induces anxiety-like behaviors and activate microglia in multiple brain regions, including hippocampus and amygdala, and accompanied with upregulated inflammatory factors (Lai et al., 2018; Cotrone et al., 2021; Zhang et al., 2021). The administration of the microglia inhibitor minocycline can significantly inhibit microglia activation in the striatum, and relieve anxiety-like behaviors (Han et al., 2020). In the present study, a single intraperitoneal injection of S-ketamine in SPS-exposed rats significantly inhibited microglial activation in striatum and PAG, and those in the ACC and PFC were not observed. Anxiety is associated with structural and functional changes in both the hypothesized fear and the limbic cortico-striato-thalamocortical circuits (Perepezko et al., 2021). Nevertheless, previous studies showed multivariate results of the cortex in PTSD models. In an early-life stress and herpes zoster pain model, microglia in the PFC is markedly activated (Ma et al., 2021; Usui et al., 2021). Neuroinflammation originating from microglia in the medial PFC induced by chronic stress, leading to depressive-like behavior (Kitaoka, 2022). Significant decreases in IL-10 and TNF-α expression and increases in the iNOS in microglial isolated from the frontal cortex of the SPS-exposed rats on Day1, but not on Day3 or Day7 (Cotrone et al., 2021). Oxidative stress is present in hippocampus and amygdala but not in cortex on Day7 after the SPS procedure (Petrovic et al., 2018). SPS exposure increased the levels of pro-inflammatory markers and microglia activation in the hippocampus and PFC on Day8 (Hong et al., 2021). However, the activation of microglia in the ACC and PFC regions, and the anxiety-like behavior mediated by SPS have not been reported. It remains controversial as to whether stress promotes or suppresses microglial inflammatory mediator release, with different results reported depending on the type of stressors, observation time point, and the brain region being measured (Enomoto and Kato, 2021). Our study showed no morphological changes of microglia in the ACC and PFC areas after SPS. However, because we only acquired measurements at Day14 after SPS, we can only draw conclusions for this single timepoint. PAG is the key brain region for the upstream pain signal transduction and descending pain regulation. In some nerve injury models, microglia activation in PAG can cause the descending pain facilitation in rats (Ni et al., 2016). Microglia activation further upregulates NF-κB and its downstream inflammatory factors like TNF-α and IL-1β (Corder et al., 2019). The accumulation of inflammatory factors leads to nerve damage, increases excitatory synaptic transmission and reduces inhibitory synaptic transmission, thus causing anxiety symptoms *via* altering signal transmission (O’Callaghan et al., 2015; Felger, 2018). In the current study, SPS induced activation of microglia in the dorsal striatum and PAG, rather than the ACC and PFC, suggesting that the response of microglia to stress exposure was attributed to the specific microenvironment of brain regions. Consistently, pro-inflammatory cytokines (e.g., TNF-α and IL-1β) and NF-κB were upregulated in those brain regions enriched in activated microglia. Previous study showed that a subanesthetic dose of ketamine could activate Bmal1, downregulate the NMDA-NF-κB signaling pathway, and reduce inflammation and microglia activation in hippocampus to alleviate postoperative neurocognitive disorder (PND) in aged mice undergoing partial hepatectomy (PH) (Niu et al., 2021). So far, there is no report that S-ketamine inhibits microglia activation through NF-κB signaling pathway in SPS model. In our study, intraperitoneal injection of S-ketamine counteracted SPS-induced microglia activation accompany with decreased levels of pro-inflammatory cytokines and p-NF-κB in the dorsal striatum and PAG.

Some limitations in the present study should be noted. First of all, inhibitors of microglia like minocycline were not used to suppress microglial activation. Although (R,S)-ketamine has been widely reported to inhibit microglial activation, we were unable to rule out its direct role on neurons in the CNS. Secondly, whether other glial cells in these brain regions were involved in the pathogenesis of PTSD and the underlying mechanisms were unclear. Thirdly, we only explored the role of S-ketamine in PTSD symptoms and it should be compared with other types of ketamine.

## Conclusion

In conclusion, our study demonstrated that S-ketamine alleviated anxiety-like behaviors and mechanical allodynia in the SPS-exposed rats, which downregulated pro-inflammatory cytokines in the dorsal striatum and PAG.

## Data Availability Statement

The raw data supporting the conclusions of this article will be made available by the authors, without undue reservation.

## Ethics Statement

The animal study was reviewed and approved by the Experimental Animal Ethics Committee of Affiliated Drum Tower Hospital of Medical Department of Nanjing University.

## Author Contributions

ZM, YL, and YH: conception and design. KX and SY: acquisition of data. XX, JZ, YJ, QL, and RX: data analysis and interpretation. YL, YH, and XG: revision for intellectual content. SY, KX, and YH: final approval of the completed manuscript. All authors contributed to the article and approved the submitted version.

## Conflict of Interest

The authors declare that the research was conducted in the absence of any commercial or financial relationships that could be construed as a potential conflict of interest.

## Publisher’s Note

All claims expressed in this article are solely those of the authors and do not necessarily represent those of their affiliated organizations, or those of the publisher, the editors and the reviewers. Any product that may be evaluated in this article, or claim that may be made by its manufacturer, is not guaranteed or endorsed by the publisher.
